# Development and characterization of a novel human CD137 agonistic antibody with anti‐tumor activity and a good safety profile in non‐human primates

**DOI:** 10.1002/2211-5463.13494

**Published:** 2022-10-09

**Authors:** Yingying Gao, Teddy Yang, Hu Liu, Ningning Song, Chaohui Dai, Yu Ding

**Affiliations:** ^1^ State Key Laboratory of Genetic Engineering, School of Life Sciences Fudan University Shanghai China; ^2^ Biologics Discovery Shanghai ChemPartner Co., Ltd. China; ^3^ Biologics Discovery Shanghai Hyamab Biotechnology Co., Ltd. China

**Keywords:** 4‐1BB, agonistic antibody, cancer immunotherapies, CD137, costimulatory receptors, PE0116

## Abstract

CD137 (4‐1BB, TNFRSF9), an inducible T‐cell costimulatory receptor, is expressed on activated T cells, activated NK cells, Treg cells, and several innate immune cells, including DCs, monocytes, neutrophils, mast cells, and eosinophils. In animal models and clinical trials, anti‐CD137 agonistic monoclonal antibodies have shown anti‐tumor potential, but balancing the efficacy and toxicity of anti‐CD137 agonistic monoclonal antibodies is a considerable hindrance for clinical applications. Here, we describe a novel fully human CD137 agonistic antibody (PE0116) generated from immunized harbor H2L2 human transgenic mice. PE0116 is a ligand blocker, which is also the case for Utomilumab (one of the leading CD137 agonistic drugs); PE0116 partially overlaps with Urelumab's recognized epitope. *In vitro*, PE0116 activates NF‐κB signaling, significantly promotes T‐cell proliferation, and increases cytokine secretion in the presence of cross‐linking. Importantly, PE0116 possesses robust anti‐tumor activity in the MC38 tumor model. *In vivo*, PE0116 exhibits a good safety profile and has typical pharmacokinetic characteristics of an IgG antibody in preclinical studies of non‐human primates. In summary, PE0116 is a promising anti‐CD137 antibody with a good safety profile in preclinical studies.

## Graphical abstract

We developed a CD137 agonistic antibody (PE0116) with good anti‐tumor activity and a good safety profile for clinical development. *In vitro*, PE0116 promotes proliferation and activation of T cells with cross‐linking. *In vivo*, PE0116 shows robust anti‐tumor potency in mouse tumor models and a manageable safety profile in cynomolgus macaques, with no appearance of elevated liver enzymes, thrombocytopenia, or neutropenia.

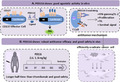

**Abbreviations**
ADCC: antibody‐dependent cell‐mediated cytotoxicity
APC: antigen‐presenting cell
CTL: cytotoxic T lymphocyte
DC: dendritic cell
MAPK: mitogen‐activated protein kinase
MHC: major histocompatibility complex
NF‐κB: nuclear factor kappa‐B
TAA: tumor‐associated antigen
TNFRSF9: tumor necrosis factor receptor superfamily member 9

In the immune response, in addition to the first signal driven by the TCR‐peptide/MHC interactions, the costimulatory signals are also indispensable to optimal T‐cell activation [1, 2, 3], the absence of which will trigger naive T cells to an anergic state [1, 4, 5, 6]. The CD28‐CD80/CD86 pathway is widely known as the most classical and unique costimulatory signal for the initial activation of T cells [3, 7, 8]. Upon the initial activation, T cells upregulate the expression of some additional costimulatory molecules, which may engage in subsequent maintenance and amplification of the immune response [1, 9]. CD137 (4‐1BB, TNFRSF9), an inducible T‐cell costimulatory receptor, is expressed on activated T cells, activated NK cells, Treg cells, and several innate immune cells, including DCs, monocytes, neutrophils, mast cells, and eosinophils [10, 11, 12, 13, 14]. Reports also suggest that CD137 surface expression is upregulated to the peak at about 60 h after stimulation and declines again by 110 h [15, 16, 17]. On T cell, the ligation with its trimeric ligand (CD137L), expressed on professional APCs [18, 19], can induce T‐cell activation, proliferation, and cytokine production by triggering downstream signal pathways and effector molecules, such as NF‐κB and MAPK, and prevent activation‐induced cell death through upregulation of antiapoptotic genes (AICD) [10, 20, 21]. Moreover, the signal preferentially activates CD8^+^ T cells, promotes IFN‐γ production, and induces robust antigen‐specific responses and generation of long‐lived memory cytotoxic T lymphocyte (CTL) [22, 23, 24, 25]. CD137 ligation promotes NK cell proliferation and cytokine secretion, and enhances antibody‐dependent cellular cytotoxicity (ADCC) function. Due to these favorable biological characteristics, CD137 engagement may provide an attractive strategy for cancer immunotherapy, functioning by restoring or boosting the natural defense against tumors by the human immune system.

In animal models and clinical trials, anti‐CD137 agonistic monoclonal antibodies have bright anti‐tumor prospects. Urelumab and Utomilumab are the two leading CD137 agonistic drugs in the clinic [26]. Urelumab (BMS‐663513, hIgG4), developed by Bristol‐Myers Squibb, is a very potent CD137 agonist and non‐ligand blocker. As expected, in preclinical studies, Urelumab can induce IFN‐γ secretion and T‐cell survival, especially improving the expansion and function of CD8^+^ tumor‐infiltrating lymphocytes and the cytolytic activity of antigen‐specific T cells [27]. But because of the liver toxicity in patients during the effective dose range, the clinical development program of Urelumab as monotherapy was terminated. In 2013, Urelumab for combination therapies entered clinical studies again [28]. Utomilumab (PF‐05082566, hIgG2), developed by Pfizer (South San Francisco, CA, USA), in contrast to Urelumab, is a ligand blocker, that requires Fc‐mediated cross‐linking to activate NF‐κB signal and promote lymphocytes proliferation and cytokine production [29]. Utomilumab has a better safety profile but is less potent against CD137 than Urelumab. Due to a good tolerable toxicity profile, Utomilumab is an excellent candidate for combination therapy [30]. EU101, a humanized IgG1 CD137 mAb, in Phase I/II clinical trials now, has strong anti‐tumor efficacy at high doses of 10 mg·kg^−1^ in the preclinical HT29 tumor model as an individual treatment. Its synergistic combination with Keytruda (anti‐PD‐1) is remarkable at low doses of 2.5 mg·kg^−1^ [31]. IBI319, a CD137/PD‐1 bispecific antibody developed by Innovent Biologics, can enhance the anti‐tumor efficacy of PD‐1 blockade without causing hepatotoxicity in CT26 and MC38 tumor models. IBI319 has demonstrated good tolerability in the first‐in‐human study of patients with advanced solid tumors or hematological malignancies [32, 33]. Balancing the efficacy and toxicity of anti‐CD137 agonistic monoclonal antibodies are considerable hindrances in clinical applications.

Here we describe the generation and characterization of PE0116, a fully human CD137 agonistic antibody based on hybridoma technology *in vitro* and *in vivo*. PE0116 has a unique binding epitope and can activate the NF‐κB signal pathway and enhance T‐cell proliferation *in vitro*. More importantly, it has robust anti‐tumor potency with a good safety profile *in vivo*. Maybe PE0116 is an attractive candidate antibody with good effects and low side effects for clinical development.

## Materials and methods

### Recombinant proteins production

The gene encoding the extracellular domain of human CD137 (UniProtKB: Q07011, Leu24‐Gln186) fused with a human Fc tag was cloned into a pCPC vector (plasmid was prepared from pCEP4, Invitrogen, Carlsbad, CA, USA), and the fusion protein huCD137‐ECD‐Fc was purified from the culture supernatant of HEK293 cells (Invitrogen) transiently transfected (PEI, Polysciences, Warrington, PA, USA) with the above plasmid. The benchmark antibodies Urelumab and Utomilumab were generated internally based on publicly available sequences. The recombinant proteins and antibodies were purified from the supernatant by protein A or Ni‐chelating affinity chromatography (GE Healthcare, Chicago, IL, USA). The molecular weight and purity of these products were verified by SDS/PAGE and mass spectrometry.

### Cell lines

The cDNA encoding the full‐length human or cynomolgus CD137 molecules were generated and cloned into the pLVX‐IRES‐puro vector (Clontech, Mountain View, CA, USA), then transiently transfected into host cells (HEK293 Invitrogen; CHOK1 and NIH3T3 ATCC). The stable cell lines (HEK293‐hCD137, CHOK1‐h/cynoCD137, NIH3T3‐hCD137) were grown and subcloned in medium suggested by ATCC with 10% fetal bovine serum (FBS, BI), 1% Pen‐strep solution (BI) and, respectively, 1, 8, 1 μg·mL^−1^ puromycin (Invitrogen) at 37 °C in the 5% CO_2_ incubator. Similarly, HEK293‐hCD137‐NF‐κB cells were constructed by transfecting plasmid pGL4.32 (Promega, Madison, WI, USA) into stable cell line HEK293‐hCD137. HEK293 cells stably expressing human CD137 and NF‐κB luciferase were routinely maintained in DMEM medium (Corning, Lowell, MA, USA) containing 10% FBS, 200 μg·mL^−1^ hygromycin (Invitrogen), and 1 μg·mL^−1^ puromycin. Intracellular expression of CD137 was analyzed by flow cytometry using fluorescence‐conjugated anti‐CD137 (BD). Luciferase expression was detected by ONE‐Glu reagent (Promega) following the manufacturer's instructions.

Stable cell line CHOK1‐OS8 displaying OKT3 antibody on the cell surface was constructed as a T‐cell activation assay stimulator. OKT3 antibody light chain variable (VL) and heavy chain variable (VH) amino acid sequences (scFv) obtained from publicly available sequences were linked with a short flexible linker [34], then fused in frame with mouse CD8a transmembrane (TM) anchoring moieties (NCBI Accession No: NP‐001074579.1, 113–220 amino acid). The fused gene was cloned into a pLVX‐IRES‐neo vector (Clontech). CHOK1 cells transformed with the fused gene expression plasmid were grown in an F12K medium with 10% FBS and 600 μg·mL^−1^ G418 (Invivogen, San Diego, CA, USA). Surface expression of the OKT3‐scFv protein on transfected CHOK1 cells was initially analyzed by FACS, stained with Alexa488‐conjugated anti‐mouse IgG antibody (Invitrogen).

### Antibody production

Chimeric antibodies were generated by, respectively, immunizing H2L2 transgenic mice (Harbor H2L2) with hCD137‐ECD‐Fc protein, HEK293‐hCD137, and NIH3T3‐hCD137 cell, and hybridoma developed as described [35]. The antibody titer in mice serum and hybridoma supernatant was measured by ELISA, flow cytometry, and luciferase reporter assay. The specific monoclonal antibodies from the supernatants cultured hybridoma cells were purified by protein G‐sepharose (GE Healthcare), containing less than 1 EU·mg^−1^ endotoxin (Charles River, Wilmington, MA, USA).

This study was performed in accordance with the protocol approved by the Institutional Animal Care and Use Committee of Shanghai Chempartner (IACUC protocol NO: A998HL0034) following the guidance of the Association for Assessment and Accreditation of Laboratory Animal Care (AAALAC).

### ELISA

ELISA 96‐well plates (BEAVER) were coated with 100 μL per well of the diluted antigen (1 μg·mL^−1^, hCD137‐ECD‐hFc), and incubated overnight at 4 °C. Block plates by adding 300 μL of Block Buffer (PBS + 1%BSA + 0.05% Tween‐20) to each well at 37 °C for a minimum of 1 h. The plates were incubated at 37 °C for 1 h after adding 100 μL hybridoma supernatants or diluted antibodies into each well, then 100 μL per well anti‐human IgG (Fab‐specific)‐peroxidase antibody (Sigma, St. Louis, MO, USA) was added and incubated at 37 °C for 0.5 h. Plates were washed three times with washing buffer (PBS + 0.05% Tween‐20) between incubation steps. Subsequently, 100 μL per well TMB was added as substrate solution (Innoreagents, Deqing, ZJ, China) and incubated for 15 min at room temperature in the dark. The reaction was quenched with Stop Solution (1 m HCl, 50 μL per well). Finally, we measured the absorbance at OD450 nm with a Molecular Devices SpectraMax Plus 384.

### Flow cytometry

CHOK1 stable cell lines overexpressing human CD137 or cynoCD137 were seeded into the 96‐well round‐bottom plates (Corning) according to 2 × 10^5^ cells per well, 100 μL per well hybridoma supernatants or serially diluted antibodies were added, and the mixtures were incubated at 4 °C for 1 h, then washed twice with FACS buffer (PBS + 2%FBS). Cells were stained with a 1 : 1000 dilution of Alexa Fluor 488 goat anti‐human IgG (Fab‐specific, Life Technology, Carlsbad, CA, USA); cells were washed twice, fixed in 0.4% PFA (Boster Biological Technology, Pleasanton, CA, USA), and analyzed by FACS Canto II flow cytometer (BD Biosciences, FranklinLakes, NJ, USA).

### Receptor blocking assay (RBA)

Flow cytometry assay was performed to evaluate the ability of anti‐hCD137 to block the binding of CD137 and CD137L based on CHOK1 cell overexpressing hCD137 and CD137L‐hFc‐Biotin (15693‐H01H‐B; Sino Biological, Beijing, China) protein. Briefly, 2 × 10^5^ cells per well CHOK1‐hCD137 or CHOK1‐cynoCD137 in FACS buffer were seeded into the 96‐well round‐bottom plates, centrifuged, and then, the supernatant was discarded. The mixture of 50 μL serial diluted anti‐CD137 and 50 μL CD137L‐hFc‐Biotin were co‐incubated at 4 °C for 1 h, then washed twice with FACS Buffer. Cells were stained with streptavidin‐Alexa Fluor 488 conjugate. Cells were analyzed by FACS Canto II flow cytometer (BD Biosciences). The IC50 value was calculated using graphpad prism5.0 (GraphPad Software Inc., San Diego, CA, USA) according to a four‐parameter fit.

### Luciferase reporter assay

A luciferase reporter assay was performed to evaluate the potency of promoting receptor signal without TCR signal using HEK293‐hCD137‐NF‐κB cell. Since some agonistic anti‐CD137 antibodies were cross‐linking‐dependent, cross‐linking antibodies were serially diluted after antibodies and F(ab')_2_ (the F(ab')_2_ is anti‐rat or human Fc domain, Jackson Immuno Research, West Grove, PA, USA) co‐incubated for 30 min at 37 °C with a mole ratio of 1 : 1.5, 1 × 10^4^ cells per well HEK293‐hCD137‐NF‐κB in 40 μL DMEM medium and 40 μL per well series diluted cross‐linking or non‐cross‐linking antibodies were seeded into the assay plates (Corning 3903) and incubated at 37 °C for 5 h. Samples were detected by a luciferase detection kit (Promega) following the manufacturer's instructions. Absorbance was measured on a microplate reader.

### T‐cell activation assay

According to the manufacturer's instructions, human peripheral blood mononuclear cells (PBMCs) were isolated from healthy donors by density gradient centrifugation on FicollPaque Plus (GE Healthcare). T cells were isolated from PBMCs following the manufacturer's protocol of the T‐cell isolation kit (Stemcell, Vancouver, BC, USA). 1 × 10^5^ cells per 150 μL assay buffer per well T cells (RPMI 1640 + 10%FBS) and 2 × 10^4^ cells per 50 μL per well CHOK1‐OS8 cells as stimulators were seeded into assay plate (Corning, 3799), 50 μL per well series diluted cross‐linking or non‐cross‐linking antibodies were added into each well. The T‐cell activation sample was incubated at 37 °C for 3 days. IFN‐γ secretion levels in culture supernatant were determined by ELISA (R&D, Minneapolis, MN, USA).

### Anti‐tumor efficacy in MC38 tumor model using CD137 knock‐in mice

The related mouse study was approved by the Biocytogen Animal Care Committee in accordance with the regulations of the IACUC. Human CD137 knock‐in female mice (6 weeks old, Biocytogen, Beijing, China, certificate number: SYXK (Jing) 2015‐0010) were subcutaneously injected with MC38 cells (5 × 10^5^ in PBS) on the right flank. Forty‐two tumor‐bearing mice were randomized into seven groups with six mice in each group until tumor volume reached about 150 ± 50 mm^3^. These mice were intraperitoneally administered with 3, 1, or 0.3 mg·kg^−1^ tested antibodies on days 0, 4, 7, 11, 14, and 18. Animal weight and tumor volumes (*V* = 0.5 × *L* × *S* ^2^, *L* and *S* are the long and short diameters of the tumor) were measured twice per week to evaluate the therapeutic efficacy of the treatment. Mice were sacrificed when tumor volume reached 3000 mm^3^, or the percentage of body weight loss exceeded 20%, and the tumor tissues were harvested and weighed.

### Pharmacokinetics and toxicity of PE0116 in Cynomolgus Monkeys

The related monkey study was approved by the Institutional Animal Care and Use Committee of Shanghai Chempartner following the guidance of IACUC protocol (NO: A998HL0051) and the Association for Assessment and Accreditation of Laboratory Animal Care (AAALAC). The cross‐reactivity of PE0116 and Utomilumab to cynomolgus CD137 is important to predict 4‐1BB‐related toxicity and pharmacokinetics. The pharmacokinetics study for PE0116 was performed together with the toxicology study. An administered group including two male and two female monkeys (3–4 years, 2.2–4 kg, certificate number: SYXK (Hu) 2015‐0021, from Hainan Jingang Laboratory Animal Co. LTD. (Hainan, China)) were intravenously injected weekly at doses of 1.6, 5 and 16 mg·kg^−1^ over 4 weeks, respectively, followed by the 2‐week recovery phase at the end. Blood samples for PK analysis were collected from individual animals on Day 0 (predose, 10 min, 1 h, 4 h, 8 h), Day 1, Day 3, Day 7 (predose and 10 min postdose), Day 14 (predose and 10 min postdose), Day 21 (predose, 10 min postdose), Day 22, Day 24, Day 28 and Day 35. Blood samples for clinical pathology (hematology, and serum chemistry) were collected from individual animals on Day 0 (predose), Day 3, Day 7, Day 14, and Day 35. The body weight of each animal was measured on Day 0 (predose) and then weekly on Day 7, Day 14, Day 21, Day 28, and Day 35, which on the day of dosing was used for dose calculation. Body temperature was monitored on the day of dosing (Predose and postdose) and Day 28, Day 35. Each animal was observed twice daily (a.m. and p.m.) for mortality and abnormalities during the whole life period. Animals would be euthanized when body weight is reduced by more than 25%, appetite loss exceeds 30% for 7 days, or animals do not respond to external stimulation. Body temperature falls < 36 °C without anesthesia or sedative. On Day 35, all animals were anesthetized and necropsied. A gross examination was performed on the organs. Liver tissue was collected and preserved in 10% NBF for future analysis.

## Results

### Antibody repertoire screening, humanization, and sequence analysis

Hybridoma clones secreting anti‐CD137 antibodies were screened by ELISA, FACS, and luciferase reporter assay. Anti‐CD137 monoclonal antibodies were purified and further characterized to determine their binding activity, specificity, and potential for activating NF‐κB signal, enhancing T‐cell proliferation, and inducing IFN‐γ synthesis *in vitro*. A rat‐human chimeric monoclonal antibody, mAb083, derived from H2L2 transgenic mice immunized by hCD137 ECD‐hFc protein, was the most promising candidate after several rounds of primary screening. To minimize the immunogenicity of the rat anti‐human CD137 antibody when administered to humans [36], the variable regions of mAb083 were sequenced and grafted onto a human IgG4 framework sequence to mediate fewer Fc‐dependent effector functions, including an engineered hinge region mutation (S228P) designed to prevent Fab‐arm exchange [37, 38]. Then, a fully human agonistic CD137 mAb, PE0116, was created. Sequence alignment of PE0116, Urelumab, and Utomilumab variable regions showed that the VH region of PE0116 shared 47% and 52% sequence homology with Urelumab and Utomilumab, respectively. PE0116 showed 68% and 54% identity in the VL region compared with Urelumab and Utomilumab, supporting PE0116 as a novel and promising therapeutic antibody (Table 1).

**Table 1:** Sequence alignment result of PE0116 with benchmark antibodies.

Variable regions (%)	Urelumab	Utomilumab
VL	68	45
VH	47	52

### Characterization and specificity of PE0116‐hIgG4

**Fig. 1:**
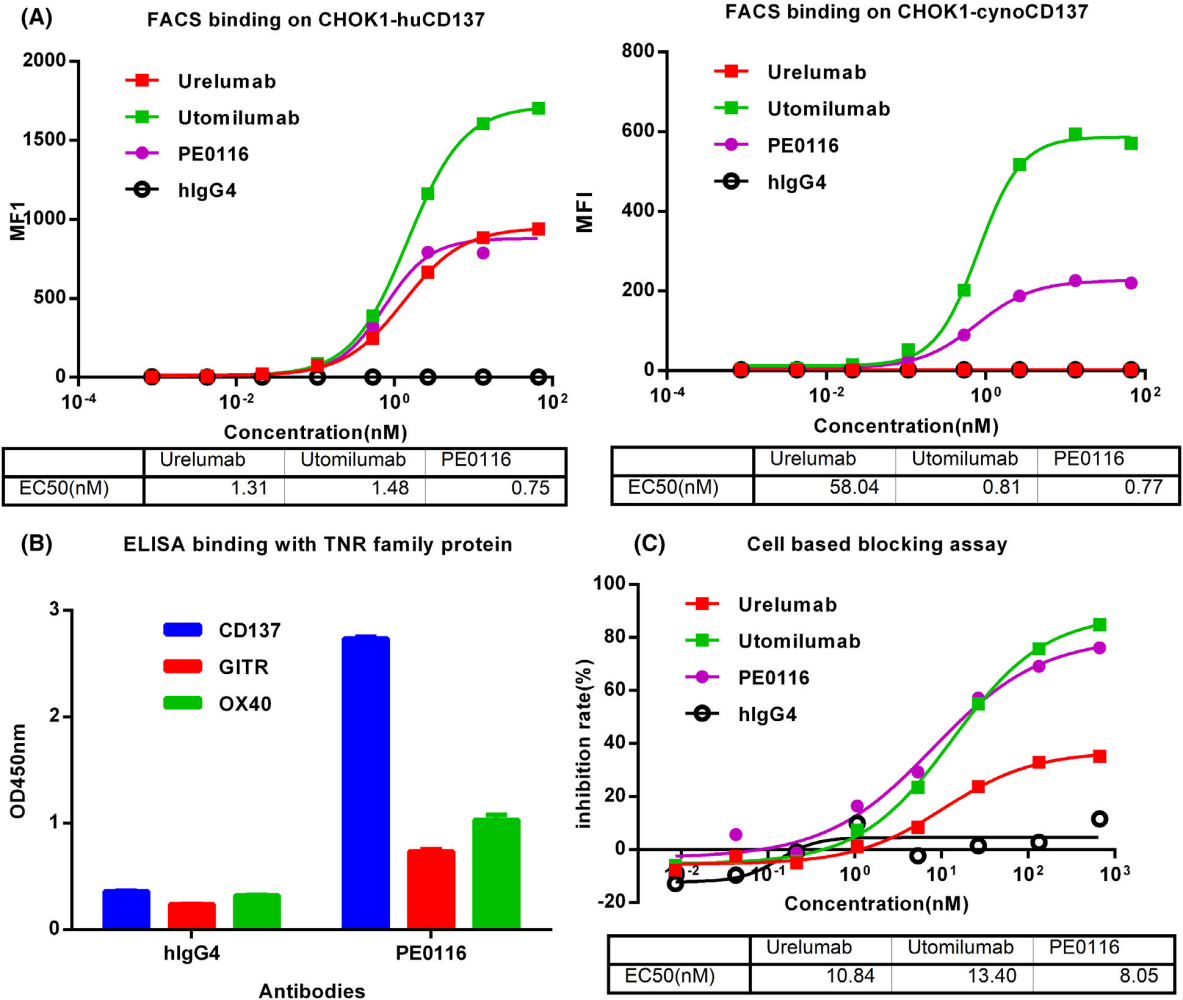
Binding, specificity, and blocking profiles of PE0116. (A) The binding activity of PE0116, Urelumab, and Utomilumab toward CHOK1‐hCD137 and CHOK1‐cynoCD137 cells was analyzed as concentration‐mean fluorescence intensity (MFI) curve. (B) Specificity of PE0116 to CD137 was conducted by ELISA after capturing 1 μg·mL^−1^ humanOX40, GITR, and CD137 as the antigen. All assays were performed in duplicate, and all error bars indicate the SD. (C) Cell‐based blocking assay was performed to evaluate PE0116, Urelumab, and Utomilumab using CHOK1‐hCD137 stable cell line and CD137L‐hFc‐Biotin with streptavidin‐Alexa Fluor 488 conjugate.

A series of assays were performed to confirm that the humanization procedure did not change the properties of PE0116. Firstly, the efficacy, pharmacokinetics, and toxicity of drug candidates were evaluated in a preclinical animal model before human use, meaning it has species cross‐reactivity [39]. As shown in Fig. 1A, the species cross‐reactivity of PE0116 was measured by FACS using CHO cells expressing human or cynoCD137. PE0116 bound to CHOK1‐hCD137 cells with EC50 of 0.75 nm, which was two‐fold superior to Urelumab (1.31 nm) and Utomilumab (1.48 nm). Like Utomilumab, PE0116 showed cross‐reactivity with surface cynoCD137, while Urelumab did not. Biacore analysis revealed the binding affinity of PE0116 to human and cynomolgus monkey CD137, with KD value of 17.6 nm (*k*_a_ = 1.68 × 10^6^ m ^−1^·s^−1^, *k*_d_ = 2.96 × 10^−2^·s^−1^) and 100 nm (*k*_a_ = 2.01 × 10^6^ m ^−1^·s^−1^, *k*_d_ = 2.02 × 10^−1^·s^−1^), respectively. An ELISA screen of related TNFR superfamily proteins (OX40, GITR) confirmed PE0116 was specifically bound to CD137 (Fig. 1B). Besides, in the cell‐based blocking assay, PE0116 was a partial blocker of CD137 and its natural ligand CD137L (Fig. 1C). Competitive ELISA shows that PE0116 shared partial epitope overlapping with Urelumab and did not compete with Utomilumab (Table 2).

**Table 2:** Competitive ELISA result of PE0116 with benchmark antibodies.

Inhibition (%)	Utomilumab	PE0116	Urelumab
Utomilumab	96	14	0
PE0116	27	92	46
Urelumab	31	94	97

### PE0116 *in vitro* functionality

**Fig. 2:**
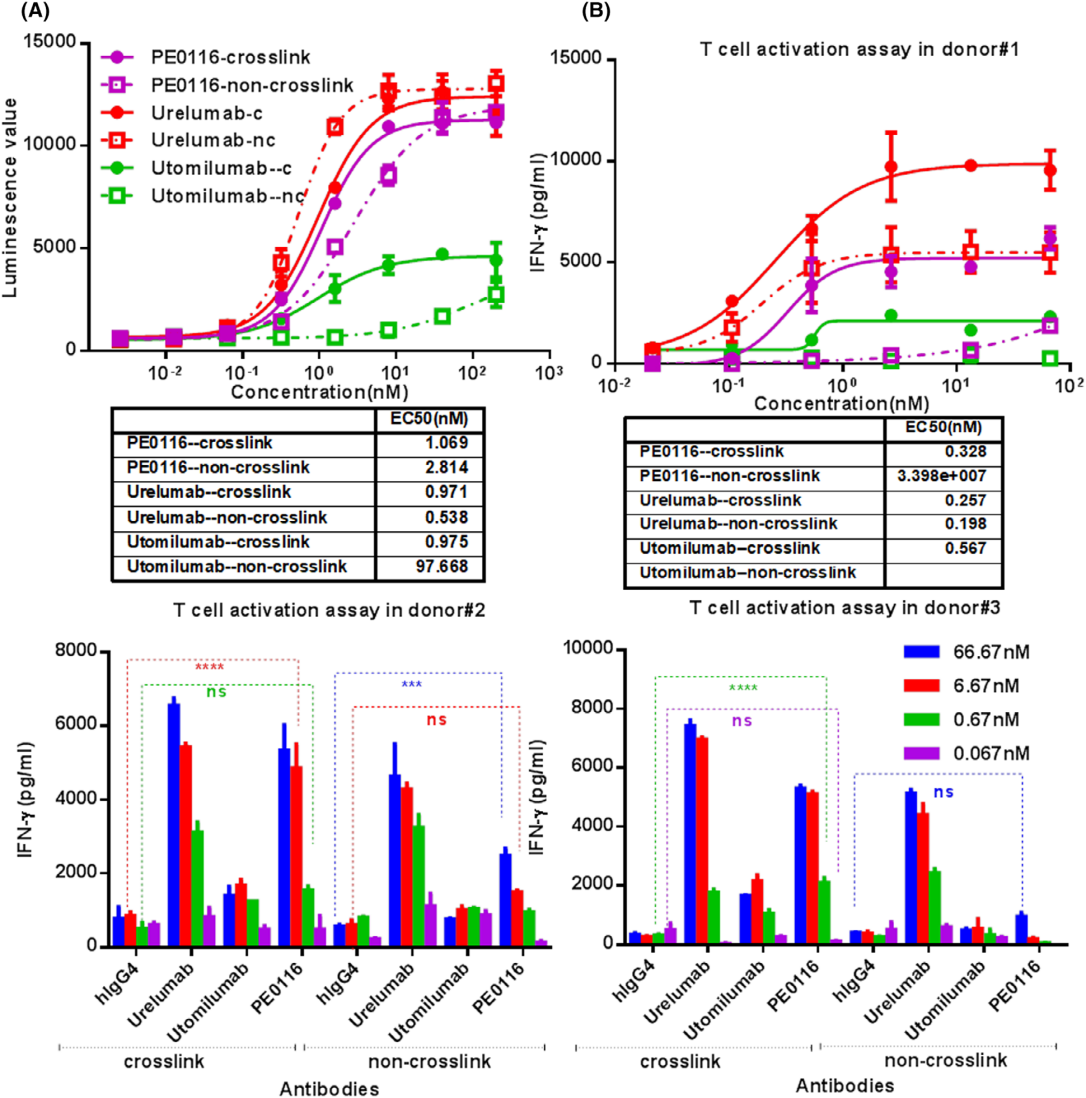
Characterization of PE0116 *in vitro* functionality. (A) Luminescence value changed by HEK293‐CD137‐NF‐κB reporter cells treated with serially diluted PE0116, Urelumab, or Utomilumab (cross‐linking or not cross‐linking, antibodies were cross‐linked by anti‐Fc F(ab')_2_ fragment, the molar ratio was 1 : 1.5). (B) Levels of IFN‐γ released by activated CD3^+^T cells from three donors after 72 h of incubation with serially diluted PE0116, Urelumab, or Utomilumab in the presence of OKT3‐scFv displayed on the CHOK1 cell surface (cross‐linking or not cross‐linking). The EC50 value of Utomilumab non‐crosslink was blank in (B) (upper right panel), representing that the dates could not be analyzed by curve fitting. ****P < 0.0001 (hIgG4 vs. PE0116 at 6.67 nm in donor#2 or at 0.67 nm in donor#3 with cross‐linking) and ***P < 0.001 (hIgG4 vs. PE0116 at 66.7 nm in donor#2 without cross‐linking), as determined by two‐way ANOVA. All assays were performed in duplicate, and all error bars indicate the SEM.

The primary mechanism involved in CD137‐targeted therapies was inducing costimulatory signals to further activate the immune system. Thus, the functional activity of PE0116 was assessed *in vitro* by the NF‐κB luciferase reporter assay. As shown in Fig. 2A, PE0116 promoted strong activation of the CD137‐dependent NF‐κB reporters in a dose‐dependent manner with about a 20‐fold increase relative to the background signal, showing almost equivalent potency to Urelumab in the presence of cross‐linking (EC50 ≈ 1 nm), and the top activation signal of PE0116 was approximately 2.5‐fold compared to that of Utomilumab. Moreover, PE0116 can efficiently enhance NF‐κB signal without cross‐linking, far superior to Utomilumab, which was cross‐linking‐dependent. In addition, a T‐cell activation assay *in vitro* was established to confirm the therapeutic potential further. Human CD3^+^ T cells were isolated from fresh PBMCs from three human donors. Similar to the behavior in the luciferase reporter assay, Utomilumab had the most insufficient agonistic activity, showing little agonistic activity without cross‐linking (Fig. 2A,B). In cross‐linking, PE0116 induced T‐cell response, leading to IFN‐γ secretion in a dose‐dependent manner, showing mighty agonistic efficiency with EC50 of 0.33 nm, nearly equal to that of the acknowledged potent agonist, Urelumab (0.26 nm). The activation strength of PE0116 was somewhat inferior to that of Urelumab in all three donors but far superior to that of Utomilumab. At the same time, PE0116 showed cross‐linking dependence in T‐cell activation, in contrast to the behavior in luciferase reporter assay, which may be a result of the CD137 expression level differences on two effector cells. The CD137 protein expressed on the cell surface of HEK293‐hCD137‐NF‐κB cells is far more than that of T cells, triggering antigen clustering for transmitting downstream signals, significantly reducing the need for antibody cross‐linking.

### Therapeutic effects of PE0116 in MC38 mouse models

**Fig. 3:**
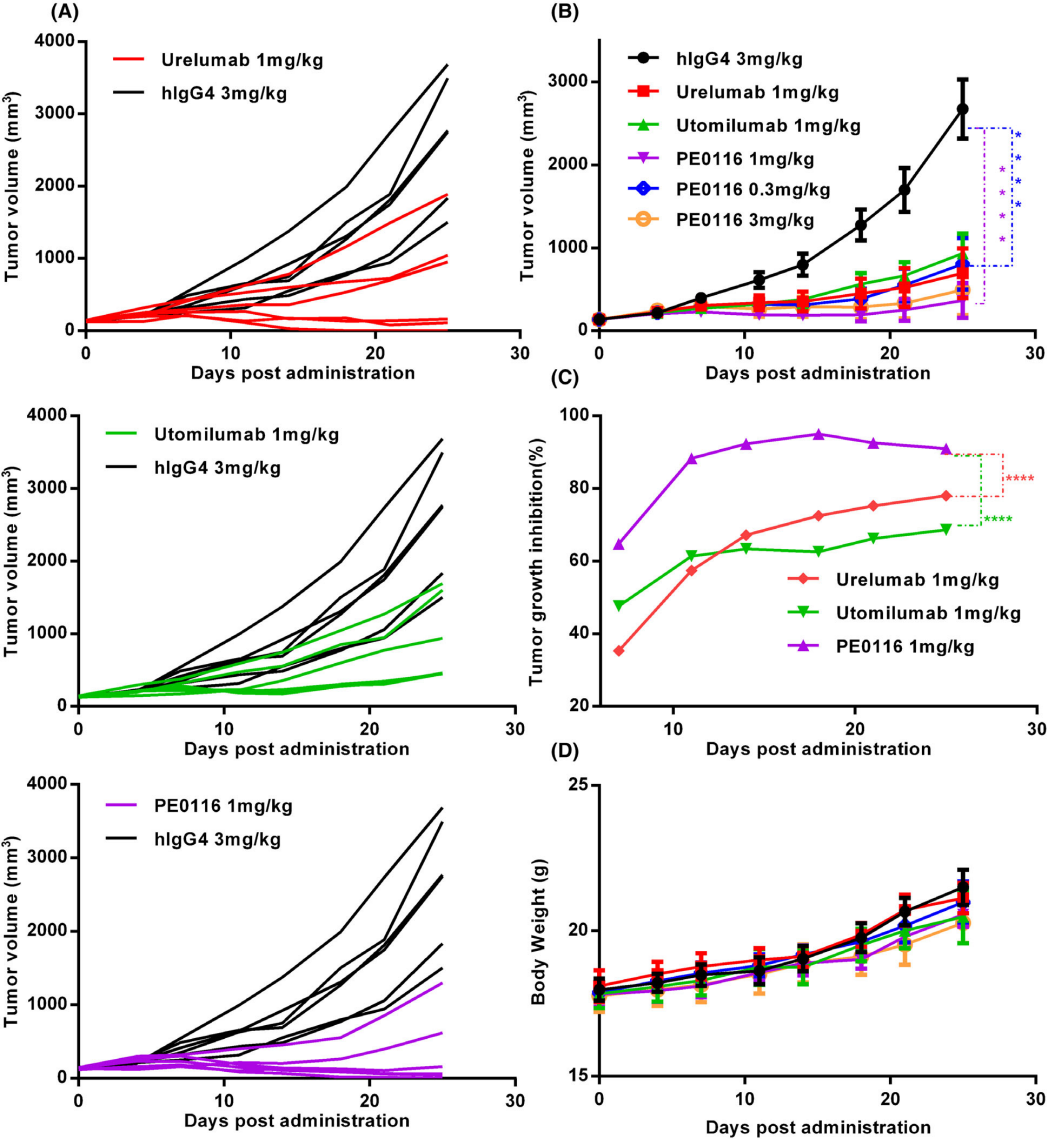
Efficacy study of PE0116 *in vivo*. (A, B) Tumor volume of mice treated with isotype‐hIgG4, PE0116, Urelumab, or Utomilumab at 1, 0.3, or 3 mg·kg^−1^ doses. Tumor diameters were monitored twice a week, and the tumor volume was estimated as 0.5 × length × width^2^. (C) The tumor growth inhibition ratio (TGI, %) of PE0116, Urelumab, and Utomilumab at 1 mg·kg^−1^. The tumor growth inhibition ratio (TGI, %) was calculated using the following formula: TGI (%) = [1 − (RTV of the treated group)/(RTV of the control group)] × 100 (%), RTV = (average tumor volume on measured day) − (average tumor volume on day 0). (D) Mice's body weight was monitored twice a week. All error bars indicate the SEM (*n* = 6). *P* values were derived using a two‐way ANOVA and compared with the control. ****P < 0.0001.

PE0116 *in vivo* activity was evaluated in a humanized mouse model using human CD137 knock‐in female mice subcutaneously engrafted with the MC38 cell line. As shown in Fig. 3A,B, PE0116 showed strong anti‐tumor activity, and sustained tumor regressions were observed. PE01116 can more effectively block tumor growth than two benchmark antibodies at 1 mg·kg^−1^ doses (Fig. 3C). For the dose‐related efficacy study (Fig. 3B), PE0116 displayed sufficiently significant anti‐tumor efficacy at 1 mg·kg^−1^ and did not further enhance tumor regression at 3 mg·kg^−1^. At low doses of 0.3 mg·kg^−1^, PE0116 still showed strong anti‐tumor potency, nearly comparable to two benchmark antibodies at 1 mg·kg^−1^ doses. There was no significant difference in the body weight between administered groups (Fig. 3D). To understand the different efficacy between Urelumab, Utomilumab, and PE0116, mechanistic studies should be performed, especially hepatotoxicity‐related parameters, including liver parameters, pathology, and liver FACS should be studied because of severe drug‐related hepatic toxicity in previous clinical studies of CD137 agonists [26].

### Pharmacokinetics (PK) and safety profile of PE0116 in cynomolgus monkeys

**Fig. 4:**
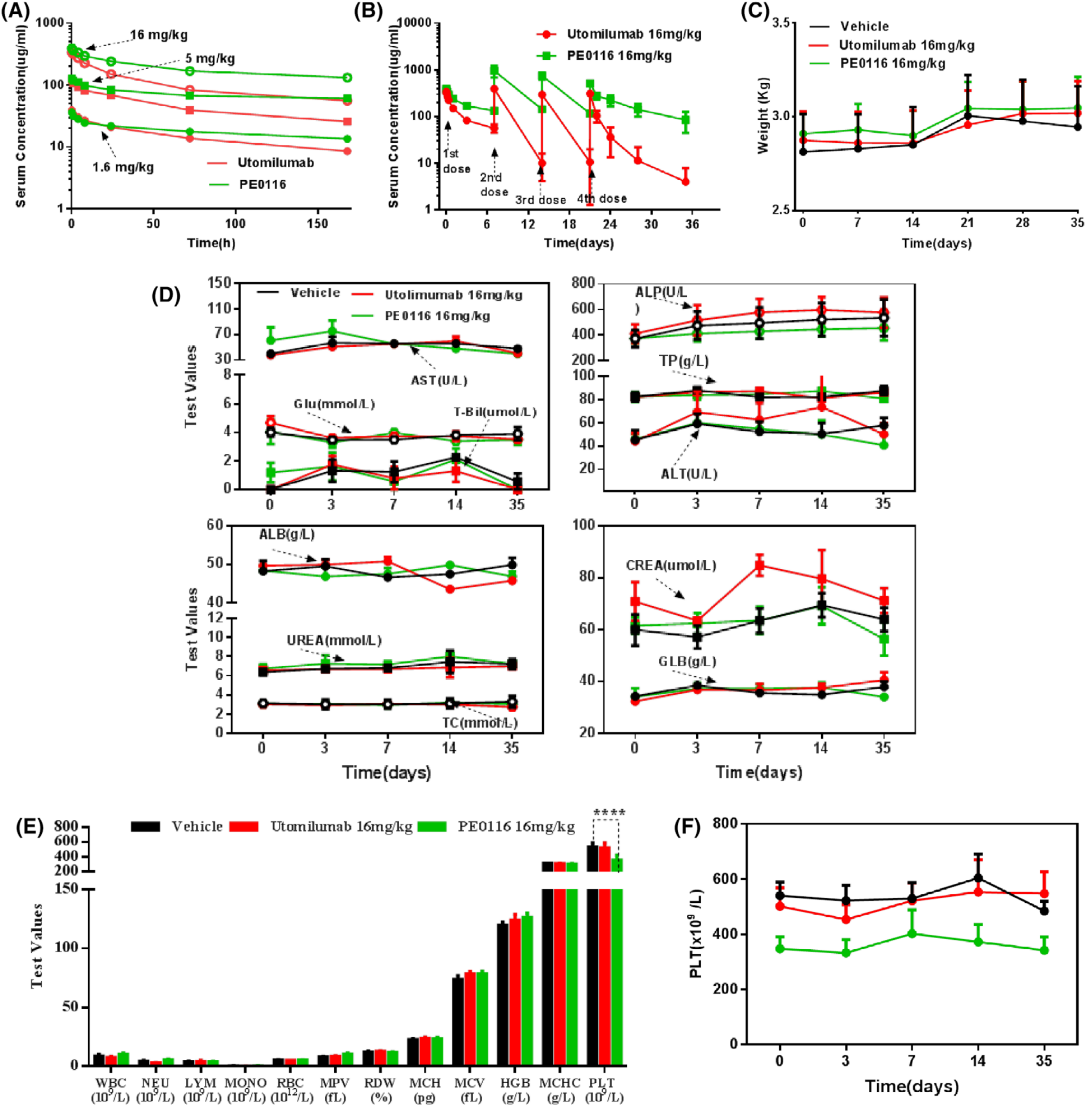
Pharmacokinetics (PK) and safety profile of PE0116 in cynomolgus monkeys. (A) Drug concentration–time curves of cynomolgus monkeys after a single i.v. administration of PE0116 or Utomilumab at 1.6, 5, and 16 mg·kg^−1^. (B) Drug concentration–time curves of cynomolgus monkeys after successive i.v. administrations of 16 mg·kg^−1^ PE0116 or Utomilumab. (C) Recorded animal weights every week in multiple 16 mg·kg^−1^ doses group. (D) Serum biochemistry levels during the 35‐day observation period in multiple 16 mg·kg^−1^ doses group. (E) Hematology levels on day 7 in multiple 16 mg·kg^−1^ doses group. (F) Platelet levels during the 35‐day observation period in multiple 16 mg·kg^−1^ doses group. All error bars indicate the SEM (*n* = 4 = 2M + 2F). *P* values were derived using a two‐way ANOVA and compared with the control. ****P < 0.0001. ALB, serum albumin; ALP, alkaline phosphatase; ALT, alanine aminotransferase; AST, aspartate aminotransferase; CREA, creatinine; GLB, globulin; GLU, glucose; HGB, hemoglobin; LYM, lymphocyte counts; MCHC, mean corpuscular hemoglobin concentration; MCH, mean corpuscular hemoglobin; MCV, mean corpuscular volume; MONO, monocyte counts; MPV, mean platelet volume; NEU, neutrophil counts; PLT, platelet counts; RBC, red blood cell count; RDW (%), percentage of red cell distribution width; T‐Bil, total bilirubin; TC, total cholesterol; TP, total protein; UREA, urea; WBC, white blood cell.

The cynomolgus monkey CD137 cross‐reactivity characteristics of PE0116 supported the cynomolgus monkey as an *in vivo* model of human pharmacodynamics and toxicity. The PK properties and toxicological effects of PE0116 and Utomilumab were evaluated in cynomolgus monkeys via cephalic vein (IV bolus) infusion at three dosages (1.6, 5, and 16 mg·kg^−1^), IV once per week for three consecutive weeks (total of four doses). Meanwhile, plasma drug concentrations, body weight, hematology, and serum biochemistry were monitored. Pharmacokinetic analysis of PE0116 showed dose‐dependent systemic exposure with PK profile and half‐life consistent standard mAb, revealing a linear relationship between clearance and dose among 1.6–16 mg·kg^−1^ (Fig. 4A). PK profile of multiple doses at 16 mg·kg^−1^ displayed that the serum exposure of PE0116 reached the peak post‐second dose, keeping a far higher level with an almost constant clearance rate during the 35‐day observation period than that of Utomilumab, which dropped sharply post‐second dose, possibly due to anti‐drug antibody occurrence (Fig. 4B). There was no significant difference in the body weight between administered groups at 16 mg·kg^−1^ (Fig. 4C). Hematology and serum biochemical tests at 16 mg·kg^−1^ were recorded on Day 0, 3, 7, 14, 35. In the serum chemistry test, slight AST elevations were caused by PE0116 on Day 3, CREA elevations on Day 7, and ALT elevations on Day 14 caused by Utomilumab appeared, and recovery to baseline levels was subsequently observed. No significant abnormalities were found for other serum chemistry parameters during the in‐life period (Fig. 4D). Based on the clinical hematological assessment results on Day 7, except for platelet index, no other treatment‐related effects were observed (Fig. 4E). The platelet levels of the three administration groups of animals were traced from day 0 to Day 35 (Fig. 4F). In the PE0116 administration groups, the platelet index was below the baseline levels before PE0116 administration, but the reason was unknown. No significant changes were found during the 35‐day observation period, which indicated that PE0116 did not induce the platelet reduction. No gender‐related differences were observed in the exposure to the tested antibody. No significant abnormalities were observed in all animals in the gross necropsy examination. No treatment‐related changes were observed in organ weights or macroscopic characteristics.

## Discussion

The strategies of immune checkpoint blockade based on immunomodulatory have revolutionized the field of immune‐oncology. However, the beneficial range of these drugs has been limited due to the immune‐related toxicities, low response rates, drug resistance, and lack of known biomarkers. There remains an urgent need to develop new strategies that enhance cancer immunotherapy and broaden beneficial patient populations [40, 41, 42]. CD137 antibodies, as costimulatory receptor agonism, have recently gained great attention.

In the present study, we have reported the generation, characterization, anti‐tumor efficacy, preliminary pharmacokinetics, and toxicology studies of a novel fully human anti‐CD137 antibody (named PE0116), which is derived from a hybridoma platform based on transgenic H2L2 mice immunized by hCD137 ECD‐hFc protein. PE0116 binds to hCD137 expressed on cell surface specifically with high affinity and shares an overlapping epitope on CD137 with its natural ligand CD137L. Moreover, its cross‐reactivity with cynomolgus CD137 allows the non‐human primate to be an *in vivo* study model for pharmacodynamics and toxicity, which is essential in preclinical drug studies. In cell‐based *in vitro* assays, PE0116 triggers NF‐κB signal pathway activation, enhances T‐cell activation, and promotes dose‐dependent secretion of anti‐tumor cytokines in response to suboptimal CD3‐TCR signal, showing mighty agonistic efficiency, which was slightly inferior to Urelumab and much superior to Utomilumab in the presence of cross‐linking. Unlike Urelumab, efficient stimulation of PE0116 on T cells is cross‐linking‐dependent. *In vivo*, PE0116 shows robust anti‐tumor activity in the MC38 colon carcinoma model. Its good safety profile is confirmed in a non‐GLP non‐human primate (NHP) study.

Clinical studies of the early two drugs, Urelumab and Utomilumab, were terminated as monotherapy because of liver toxicity and low efficacy, respectively [26], indicating that the clinical potential of agonistic antibodies is influenced by various factors, including the binding epitope, affinity, potency, cross‐linking requirements, Fc effector function and the ability to block receptor‐ligand binding. Unlike characteristics of ideal antagonists by high affinity and ligand blocker, there are no rigid rules for the design of agonistic antibodies. It is necessary to comprehensively consider and balance the above factors to ensure the optimal efficacy and safety of antibodies in the clinic, which is a significant challenge in developing CD137 agonistic antibodies [43].

It is reported that human CD137L and Utomilumab mainly combine with CRD III (cysteine‐rich domain) of CD137 receptor and the junction between CRDs III and IV, respectively [44, 45]. Urelumab binds on membrane‐distal CRD I of the CD137 receptor, where it is likely maximally exposed on the cell's surface to more permissive clustering and cross‐linking, yielding a better‐stimulated pathway than the membrane‐proximal epitope, explaining the differences in agonistic potency between Urelumab and Utomilumab. What is more, the hepatotoxicity of CD137 agonist is supposed to be due to the mechanical differences in their agonistic activity or CD137 binding properties [45, 46]. As a ligand blocker and epitope competitor of Urelumab, we hypothesize that PE0116 recognizes a unique conformational epitope between CRD1 and CRDIII of the CD137 receptor, further supporting its novel characteristics compared to Urelumab and Utomilumab, such as optimal but not most potent functional agonist properties to prevent hepatotoxicity induced by T‐cell hyperactivation.

Targeting tumor‐associated antigen (TAA) to locate the agonist exposure in the tumor microenvironment (TME) or exploiting cross‐linking‐dependence of agonist or the characteristics of TME to make agonist activated in tumor and inactivated in normal tissue are essential strategies to circumvent the systemic toxicity of agonistic antibodies in clinical development. CD137 agonist bispecific antibodies or combination with other drugs have raised high expectations, especially immune checkpoint inhibitors [47, 48]. Conceptually, delivering two immune effector activation signals of overcoming inhibition and costimulation, and restricting agonists in tumors by targeting TAA, are considered high potency and safety. Later clinical trials also prove this. For example, clinical trials of GEN1046 (bispecific antibodies PD‐L1 × CD137, BioNTech, Mainz, Rheinland‐Pfalz, Germany) show potent anti‐tumor activity *in vivo* superior to PD‐L1 blockade monotherapy and significant early clinical activity across different dose levels in the population resistant to prior immunotherapy or typically less sensitive to an immune checkpoint inhibitor in Phase I/IIa clinical trials; expectedly, a good safety also is observed [49]. We have demonstrated that PE0116 induces dose‐dependent IFN‐γ secretion in the presence of cross‐linking, and the stimulation signals conducted by PE0116 are slightly inferior to Urelumab and far superior to Utomilumab, further confirming the previous epitope hypothesis. PE0116 shows very weak T‐cell activation without cross‐linking. Through rational design strategies, the cross‐linking‐dependent activity of PE0116 is contributed to limiting the agonistic signals in tumors and significantly reducing the systemic side effects, such as achieving cross‐linking by another arm of PE0116 bispecific antibodies binding to TAA, which will be the optimization direction of PE0116 in future. Besides, PE0116 has been engineered on an IgG4(S228P) backbone to lack Fc‐dependent effector function, helping effectively to reduce hepatotoxicity.

*In vivo*, our results suggest PE0116 can induce sustained tumor regressions and show a much superior tumor growth inhibition rate at 1 mg·kg^−1^ to two benchmark antibodies in the MC38 colon carcinoma model. In addition, PE0116 performs a manageable safety profile in cynomolgus macaques, with no appearance of elevated liver enzymes, thrombocytopenia, or neutropenia, which are frequently reported as severe adverse events in clinical studies of Urelumab [50]. PE0116 shows slower clearance and consistently higher serum exposure than Utomilumab in pharmacokinetics studies, reducing the dosage and frequency of administration, possible side effects, and patients' treatment burden in clinical application, explaining that PE0116 shows more robust anti‐tumor potency than Utomilumab.

We have developed a fully human CD137 monoclonal antibody PE01116 with specificity, efficacy, and safety profile *in vitro* and *in vivo*. The encouraging preclinical characterization supports the clinical development of PE0116, and a first‐in‐human study has been registered (CXSL2000070). Although combination strategies and bispecific antibodies strategies with other drugs need further studies, our results suggest that PE0116 is an ideal component of a combination strategy.

## Conflict of interest

The authors declare the following financial competing interests: YG, HL, NS, and TY are full‐time employees of ChemPartner. CD is a full‐time employee of Hyamab. Patents for the results presented in the paper have been filed under the Patent Cooperation Treaty (International Publication Number PCT/CN2018/114641). YD declares no conflict of interest.

## Author contributions

NS, CD, and YD designed the experiments. YG and NS performed the experiments and analyzed the data. YG wrote the manuscript. YD reviewed and edited the manuscript. HL and TY supervised the project.

## Data Availability

Data are available from the corresponding author upon reasonable request.
